# Laundry and textile hygiene in healthcare and beyond

**DOI:** 10.15698/mic2019.07.682

**Published:** 2019-07-01

**Authors:** Dirk P. Bockmühl, Jan Schages, Laura Rehberg

**Affiliations:** 1 Rhine-Waal University of Applied Sciences, Faculty of Life Sciences, Hygiene and Microbiology, Marie-Curie-Str. 1, 47533 Kleve, Germany.

**Keywords:** laundry, hygiene, microbiological contamination, healthcare, biofilm, washing appliances

## Abstract

The elimination of microbial contaminations from textile is an important aspect of laundering apart from the removal of stains and dirt from used and worn textiles. Although the framework for institutional laundering is well regulated to ensure hygienic cleanliness via the use of e.g. high temperatures and bleaching agents, there are several open points, especially in domestic laundering. In both cases, energy efficiency of appliances is a main driver for innovation and has resulted in a general decrease in washing temperatures which in turn can impact the antimicrobial efficacy of laundering. Thus, the different factors influencing the input and removal of microbial cells in the laundering process and possible adverse effects of microbial contaminants in the washing machine and on the textiles as well as suitable counteractions are discussed in this article, focusing on the clinical area but also considering the domestic environment, which will gain importance in the future, e.g. by the increase of elderly and ill persons being cared for at home.

## INTRODUCTION

The main task of laundering is the removal of visible stains and soils, which can be determined visually by the professional and non-professional consumer. However, the washing procedure should also lead to a hygienically clean textile surface, which includes the reduction of microorganisms on the fabric to a level safe for use as well as addressing other adverse microbial effects, e.g. malodor formation. Traditionally, this issue was solved by a combination of time, temperature, mechanics and chemistry, acting together as a means to either remove the microbial cells or kill them. In this regard, oxidizing compounds, such as chlorine or activated oxygen bleach and temperatures of or above 60°C play a crucial role to ensure an efficient antimicrobial action of the laundering process.

In the past years especially the use of higher temperatures was aimed to be limited, thereby reducing the energy consumption but also taking away a reliable method to control microbial contaminations. As a consequence, it is necessary to gain a deeper understanding of microbiological problems and possible counteractions to be sure to obtain a proper level of hygiene.

There are two major aspects within the laundering process from a microbiological point of view. First, the removal or inactivation of pathogens on either washables or the washing devices itself has to be considered. This refers to cross-contamination on the one hand, i.e. textile to textile or washing machine to textile and vice versa, as well as to an insufficient reduction of an existing bioburden. The second aspect includes impairments with a lesser risk of infection, such as biofilm formation inside the washing machines and the re-contamination of already washed textiles during rinsing cycles and malodor formation (either on the textiles or in the washing machine) as a consequence.

**Figure:**
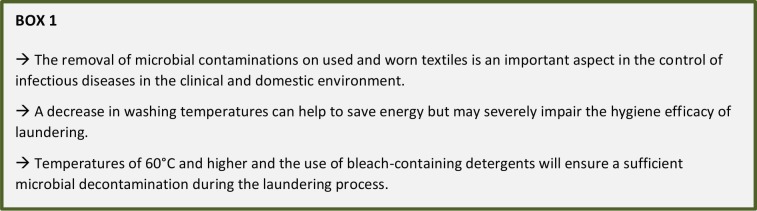


## INFECTION RISK ASSOCIATED WITH LAUNDRY

**Figure 1:**
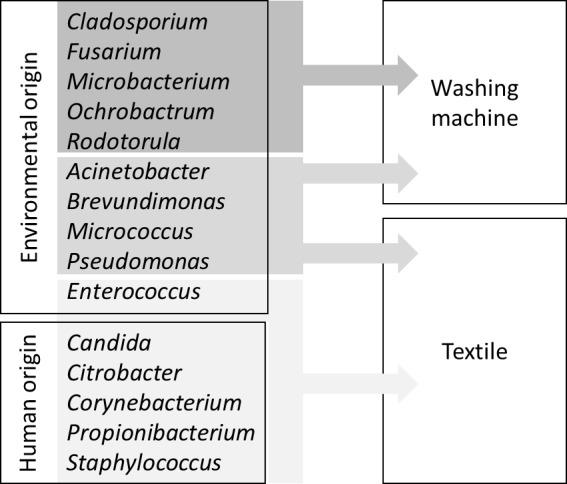
FIGURE 1: Selected microorganisms found on textiles or in washing machines and their possible origins (adapted from [14]).

Microbial contaminations on laundry items or textiles can originate from different sources. Textiles undergo numerous stages during the utilization cycle, i.e. wearing, laundering, drying, ironing and storage, which are related to different species of microorganisms and different contamination pathways. Many microbial species are transferred to textiles via skin contact by wearing the laundry items. For example, members of the human skin and mucosal biota can typically be brought to clothes and towels with direct body contact [1]. In addition, contaminations can originate from bodily excretions. Items such as underwear contaminated with excretions or shirts soiled with microbial species from the armpits are might thus transfer a quite characteristic microbiota to the textile after contact, even though other members of the skin microbiota can also be assumed, but in a lower extent. Finally, it has to be considered that other types of textiles, such as cleaning textiles, bed linen or surgical textiles may be contaminated in additional ways. An overview presenting selected microorganisms isolated from textiles and/or the washing machine can be found in **Figure 1**.

The origin of microbial contamination on textiles is closely linked to their use in different environments and includes dust, soil and food, which might even point to specific areas, e.g. healthcare facilities. Some studies consider a contamination with microorganisms resistant to desiccation (for example *Staphylococcus aureus*) as typical for this environment and assume that the amount of bioburden on laundry items like towels or sheets (depending on certain situation-dependent factors) might be up to 10^4^-10^6^ cfu/cm^2^ [1–3].

While usually one purpose of the washing process is the removal and inactivation of microorganisms on the fabric as well as the cleaning of laundry items, it has been shown that the washing machine can be considered as a source of re-contamination of textiles [4]. As mentioned above, biofilms could be a potential reservoir for pathogens and might detach during the washing process and re-contaminate the laundry [5–7]. The composition of the microbiota on textiles and in the washing machine has been subject to several investigations [2, 8–12]. In this regard it can be assumed that the washing process may cause a shift in the microbial community on textiles form primary contaminants (skin bacteria) to secondary contaminants (biofilm-associated environmental bacteria) and that the water itself can also be a source for recontamination and contribute to secondary contaminations [10, 13, 14].

The generation of malodors on textiles caused by the skin microbiota as well as microorganisms colonizing the washing machine might be considered as another microbiological problem. This aesthetic impairment is connected to two typical laundry malodors: a musty “wet-cloth-like” odor and acidic, sweat like odors [15–19]. Munk *et al.* (2001) and Kubota *et al.*(2012) postulated that the formation of smelly substances on washed textiles is caused by bacterial metabolization of sweat residues, for example by *Moraxella* species [19–22]. Most likely branched, unsaturated fatty acids like 4-methyl-3-hexenoic acid (4M3H), which have this “wet-and-dust-like” odor and are also present in human sweat after bacterial metabolization, might be responsible for the malodor [23–25], but neither the compounds which are responsible for the malodor nor the exact pathway of their formation are understood in detail. Since a wide range of microorganisms has been identified from biofilms and other areas of European washing devices [8, 11, 26], the formation of laundry related malodors and biofilm formation within the washing machine might be linked and deserves increased attention.

There are several studies reporting outbreaks related to contaminated laundry, mostly associated with bacterial pathogens, although also viruses and fungi may play a role [1]. In general, it can be assumed that most of the microorganisms found on textiles should not pose a considerable health risk, as long as these microorganisms are part of either the transient or the resident human skin flora which we are in permanent contact anyway. However, studies showed that in case of infections with i.e. *S. aureus*, MRSA (multi-resistant *S. aureus*) or *Clostridium difficile*, the pathogens were often present on the clothing of both healthcare workers and patients and bed linens as well. Other species, such as *Pseudomonas aeruginosa* or *Trichophyton mentagrophytes* (athlete's foot) from textiles could be associated with infected patients as well [1, 27]. Outbreaks in clinical settings attributed to textiles have been frequently caused by *Bacillus cereus* but also by *Acinetobacter spp.* or *Aspergillus flavus* [28]. Since those species are environmental pathogens, a contamination might have occurred after or during the laundering process. Therefore, in clinical settings or some situations a potential health risk from laundry should be taken into account, especially when immunocompromised persons are affected. This is of a great concern for healthcare facilities like hospitals but also regarding the increasing number of people being cared for at home. Inadequate laundry hygiene can be a problem for these risk groups including infants, elderly people, pregnant women and, as mentioned before, people with a deficient immune system. In this case, a reinfection by insufficiently decontaminated textiles as well as the transmission of infections among members by cross-contamination might cause major problems. One of the most relevant aspects of washing machines in the clinical environment might be their role in harboring (and possibly spreading) water-borne, multi-resistant bacteria, which have to be considered a rising threat [29]. In hospitals, healthy carriers of MRSA or other resistant strains like ESBL (extended spectrum beta-lactamase)-producing species might transfer these pathogens to other patients or infect themselves during a hospital stay with their own resistant bacteria. This silent spread of antibiotic-resistant strains poses an increasing health risk in the hospital as well as in the community sector [27].

## LAUNDRING AS AN ANTIMICROBIAL PROCESS

According to Sinner (1960), the cleaning performance of a washing process is determined by four variables: temperature, mechanical action, chemistry and time (duration) [30]. Nevertheless, a lot of parameters which are not mentioned within Sinner`s principle might influence the antimicrobial performance of a laundering process as well, e.g. the occurring microbial species, the amount and kind of soil as well as the embedding matrix or the quality and quantity of a contamination.

The temperature of a washing process has various functions: it affects the microbial reduction on laundry items by thermal inactivation, it accelerates the activation of chemical additives such as bleach and it facilitates the mechanical removal of soil. Temperatures of 60°C or above are known to inactivate microorganisms and thus ensure a high level of hygiene in laundry, and consequently are widely used in the institutional sector. In order to save energy a trend towards lower temperatures can be observed, yet this might decrease the antimicrobial performance of a washing process. Following the Sinner's principle, a decreased temperature can be compensated by the increase of one or more other variables (e.g. by extending the wash cycle time). It could be shown that for cold temperatures a longer washing cycle would not completely restore the antimicrobial performance of laundering, while increasing the chemical part, e.g. by using bleach containing detergents might better compensate for the lack of temperature [31].

As mentioned, the microorganisms present will also highly influence the microbial reduction, because killing bacteria like *Enterococcus faecium* needs high temperatures, while others may be more prone to chemical inactivation or mechanical removal. Studies suggest that in general temperatures above 50°C might be able to significantly reduce a wide range of microorganisms on textiles, even without the use of bleach containing detergents [13, 27, 31–39].

The type and construction of the washing machine has to be considered as one of the major factors influencing the mechanical action of laundering. In European households and the whole industrial and institutional sector, horizontal axis washing machines are mainly used, while in North America and Asia vertical axis washing machines are common as well. It must be assumed that the mechanical effect of a washing machine contributes to the microbial reduction on the textile, although this assumption is mainly supported by laboratory data by now. Nevertheless it could be shown by Honisch *et al.* that in washing cycles with low temperature and low amount of detergent and additive (without bleach) the mechanic might be an important influence by physically removing cells from textiles [40]. Apart from the construction type of the device other factors such as the liquor ratio might affect the microbial reduction.

Chemistry as an important factor influencing the antimicrobial efficacy of laundering processes refers to the effect of detergents and laundry additives, with bleaching agents, quaternary ammonium compounds and surfactants being the driving forces of antimicrobial efficacy. Surfactants account for the cleaning efficacy of the laundering process by removing hydrophobic soil and therefore improve the physical removal of microbial cells from the textiles as well. This effect, rather than an antimicrobial effect might account for higher microbial reductions in presence of surfactants [40, 41].

In contrast, bleaching agents are presumably the most important component determining the antimicrobial activity of the laundering processes. While in America or Southern Europe chlorine bleach has been used traditionally, in Western and Northern Europe activated oxygen bleach (AOB) predominates. AOB is based on perborate or percarbonate, which can release hydrogen peroxide in aqueous solutions. Since this effect requires higher temperatures, bleach activators such as TAED (tetraacetylethylenediamine) are used to induce the formation of peracetic acid, which happens even below 60°C. Peracids can be formulated into solid detergent, thus providing a high microbial reduction during laundering. Various studies demonstrate that the use of AOB significantly increases the antimicrobial efficacy, but the amount of the microbial reduction also varies depending on the tested microorganisms and the used conditions [31, 35, 38].

Besides AOB or chlorine bleach, the use of quaternary ammonium compounds (QAC) like benzalkonium chloride (BAC) and dimethyl didecyl ammonium chloride (DDAC) can be used to increase the antimicrobial activity of laundering. Considering the fact that anionic surfactants are widely used in laundry detergents and the cationic QACs are not compatible with those, QUACs are particularly used during rinsing after the main wash cycle. QACs can interact with the surface of negatively charged textiles, leading to the presumption that those compounds may stay on the textiles even after laundering, thus providing a persisting antimicrobial effect. Studies suggest that microbial contaminations of laundry might be mediated by biofilms inside washing machines [4, 13] and that QACs containing products might be able to compensate this effect [39]. Still, the exerted antimicrobial efficacy highly depends on the type of microorganism, so the use of QACs results in a high reduction of gram-positive bacteria even at low concentrations, whilst for the inactivation of fungi or gram-negative bacteria higher concentrations are required [42]. When using rinse aids containing QACs it must be considered that these compounds may enhance resistances against biocides and may even support cross-resistance to antibiotics [43, 44].

## EVALUATING AND MONITORING THE ANTIMICROBIAL PERFORMANCE OF LAUNDRING

As mentioned before, laundering as a means to reduce the microbial bioburden on textiles must be considered a combination of removal and inactivation. The currently available standard procedures to measure the antimicrobial efficacy of laundering use artificially contaminated swatches and textiles. Most of these investigations are performed in washing machines, since alternative methods such as suspension tests do not allow for a precise simulation of washing processes due to the lack of mechanical impact [45, 46].

Industrial and institutional laundry disinfection processes are normally relying on a combination of high temperatures and antimicrobial chemistry, mainly comprised of liquid detergents and disinfectants. This process has to be considered different from solely chemical or thermal disinfection processes, because its efficacy is affected by more than one parameter. In many countries the healthcare sector is strongly regulated by governmental authorities, some of which have also published rules and methods for evaluating the antimicrobial performance of laundering processes. Methods for testing chemo-thermal laundry disinfection are described inter alia in the European standard EN 16616 using a vertical or horizontal-axis machine while some American standards such as ASTM E2274 or ASTM 2406 use lab-scale devices [47–49] It must be considered that these kind of test aim to show the principle efficacy of a disinfection process while examinations of running processes might be advised to be tested by other means, as described below.

All mentioned procedures determine the antimicrobial efficacy using cotton swatches immersed with specified total viable counts (TVC) of different microbial species like *S. aureus*, *Candida albicans* or *Escherichia coli*. The prepared carriers are laundered together with ballast textiles and a predetermined amount of organic soil [47]. Other test methods like IEC/PAS 62958 and DIN EN 60456 are mainly dealing with the machine equipment itself with the latter predominantly focus on the cleaning performance [50, 51]. By comparing the initial microbial load and the remaining TVC after the laundering process (i.e. the disinfection step) the efficacy of the microbial reduction is determined and can be expressed in a (logarithmic) reduction factor. This evaluation is done using standardized methods such as decimal solution series and microbial surface culture [52]. As mentioned above, the cross-contamination of textile is another important aspect regarding the efficacy of a laundering process. This effect can be detected using pre-sterilized pieces of cotton swatches which are washed together with the artificially contaminated swatches and the textile load as control.

Apart from this normative methods, studies with naturally contaminated laundry items have been performed to investigate the antimicrobial effectiveness of laundering processes either in the institutional sector or in domestic environments [2, 4, 53]. Even though these investigations can be considered more realistic, there are some limitations within these approaches. For example, the detection of the microbial reduction achieved by the laundering of naturally soiled items is limited, because the highest detectable reduction of the microbial load depends on the initial contamination, which is normally lower than for standard tests. Furthermore, the composition of soils and microorganisms is unknown. Thus, the upper detection limit with naturally contaminated laundry items may be too low for an accurate evaluation of the hygiene efficacy of washing processes, since an initial load of approximately 10^4^-10^6^ colony forming units (cfu) per mL is necessary, to detect the reduction minimally required for disinfection.

As mentioned above, the European standard EN 16616 focuses on chemical-thermal textile disinfection for areas in which a disinfection is required, for example hospitals or food processing premises. This method requires the investigation of bacteria (*E. coli*, *S. aureus*, *P. aeruginosa* and *Enterococcus hirae*), yeasts (*C. albicans)* and mold (*Aspergillus brasiliensis*). If applicable, mycobacteria can be tested in addition for laundry processes using temperatures below 60°C while *E. faecium* should be tested for processes above 60°C [47]. Although this variety of microorganisms is believed to be representative for most areas and situations and must also be manageable test strains comprising the range of resistance against temperature, time and chemicals, other microorganisms might be added to consider different conditions, for example regarding the domestic area.

However, it might not be possible to evaluate the antimicrobial efficacy of a laundering process comprehensively based on these microorganisms only, because of the more diverse nature of microbiota in real life and the varying impact of factors like program duration, temperature or detergents. Still, it can be assumed from existing studies that except for heat-resistant strains, such as *E. faecium,* most bacteria are inactivated quite well even at lower temperatures when bleach is used [31, 35, 38]. Laundering without bleach appears to be somehow effective against gram-negative bacteria, perhaps due to the presence of an outer cell membrane that might be more prone to detergent attacks [14, 31]. Bacterial spores are known to be more resistant than vegetative cells and have to be considered as relevant contaminants especially in healthcare facilities. Here, spore-forming bacteria such as *C. difficile* might pose a problem. Those species have already been isolated from the bed linen of patients with a positive stool toxin test even after laundering at 71°C [53].

Infections related to viral pathogens such as enteric viruses (norovirus and rotavirus), respiratory viruses (influenza) and herpesvirus as well as poliovirus commonly occur in everyday life and healthcare settings. To effectively inactivate viruses during laundering is crucial, since textiles might act as vectors in the chain of infection[1]. One of the most important aspects regarding the virucidal effectiveness of laundering seems to be the outer structure of viral particles. Some studies suggest that laundering is more effective against enveloped viruses due to the phospholipid envelope which can be disturbed by the detergent [54–58]. For non-enveloped viruses (e.g. Norovirus) it has been shown that AOB substantially improves the antiviral efficacy of the laundering process too, yet only at temperatures above 60°C a complete inactivation can be assured [58, 59].

Finally, the antifungal efficacy of a laundering process is very important, because fungal infections are very common and the textiles may serve as vectors here, too. The infectious dose of dermatophyte fungi is very low and it has been shown that socks worn by patients with dermatomycoses can carry huge amounts of fungal cells and spores, which might be a potential transmission route [37]. Again, in experimental studies investigating the elimination of fungal pathogens such as *Trichophyton* and *Candida* from contaminated textiles it could be shown that AOB and higher temperatures might provide a nearly complete inactivation [31, 37].

## LAUNDRY HYGIENE IN HEALTHCARE

**Figure 2:**
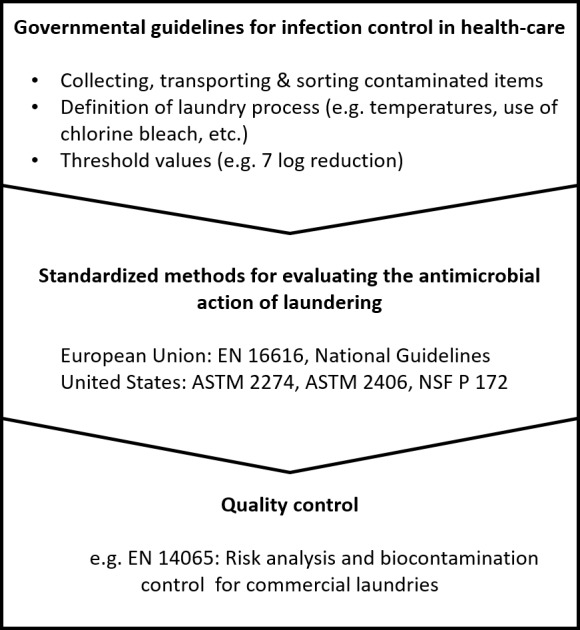
FIGURE 2: Defining and controlling laundry hygiene in health care facilities.

For healthcare facilities as areas of higher risk, the prevention of the transmission of pathogens is a major element of an infection prevention program. Cross-contamination via textiles is considered to be a transfer route for pathogens, thus, authorities such as the German Robert-Koch-Institute (RKI) - for authority ordered disinfection -, the German Association for Applied Hygiene (VAH) for quality assurance in prophylactic disinfection or the Center for Disease Control and Prevention (CDC) in the US periodically publish recommendations on how laundry and textiles in hospitals or other healthcare facilities should be handled in this areas (**Figure 2**). These recommendations are adjusted to the special risk and the field of application. For example, laundry for the surgical area needs to fulfill highest requirements and must be sterile, while in other areas the level of decontamination may be lower. According to the RKI other laundry items only have to be free of vegetative pathogens, verified by no growth of more than two colonies per 10 cm^2^ of the textiles on contact plates, followed by specific investigations. In cases of highly contagious infections (anthrax, cholera), disposable textiles are recommended while for infections like hepatitis A or diphtheria, accepted disinfection measures like thermal and chemical methods should be performed [60]. The CDC recommends laundry temperatures of at least 71°C for 25 minutes regarding hot-water-laundering, which provides a microbial reduction of at least 5 log steps per cm^2^, or the addition of a bleach agent if low temperatures are used [28, 61]. Although laundry in health care facilities should be hygienically clean, thus carrying no risk to health-care workers and patients, no microbiological definition of the CDC exists [28]. Likewise, a regular examination of the antimicrobial efficacy of the laundering process is recommended and further controls and examination might be necessary in case of exceeding guideline limits. For example, the handling of dry and wet laundry, the influence of drying and the determination of the microbial contamination on e.g. hands, surfaces and additional textiles have to be regarded [62].

In Europe, a quality management system of “Risk Analysis and Biocontamination Control” (RABC) is described in the standard EN 14065 aiming to improve the prevention of microbial contaminations on persons or products within the laundering process and utilization cycle to assure a defined microbial quality of textiles [63]. In this standard no thresholds for microbiological contaminations are defined, so a validation for all hygienically challenging areas, in particular healthcare facilities and the food sector, is needed. Like in other normative methods the tests according to EN 14065 are performed with contaminated swatches to evaluate the antimicrobial efficacy of the process [64].

Although a lot of healthcare activities have nowadays been moved from hospitals to the domestic environment, the required level of hygiene in domestic laundering is not defined. It can be assumed that neither a special disinfection nor a sterilization is necessary for domestic laundering processes, as far as healthy persons are concerned. Nevertheless, the washing behavior in households has been changing due to the ongoing trend towards lower washing temperatures, while the number of people at higher risk being cared for in a domestic environment might even increase in the future. Especially when avoiding high temperatures in order to save energy for the sake of sustainability, it has to be considered that the microbiological quality of the laundry might decrease [65].

Establishing minimum hygiene requirements for domestic laundering might be a challenge, because there is still a lack of knowledge about the extent of contaminations and the composition of pathogens or resistant microorganisms on the textiles.

## CONCLUSION

Considering the laundering process, it is crucial to be able to control the transmission of infections in healthcare facilities as well as domestic environments. Inactivating or removing microorganisms from textiles achieved by means of temperature, detergents or mechanical action can help to break the chain of infection. Since the majority of microorganisms found on textiles are also part of the human microbiome or the environment, they mostly should not pose human health risk. Nevertheless, whenever a sufficient level of hygiene must be guaranteed, laundering at higher temperatures (i.e. 60°C) and the use of bleach is recommended. This is especially important in critical cases such as acute infections or if special risk groups are affected. Therefore, the trend towards lower washing temperatures in the attempt of saving energy costs can impair the microbial reduction during laundering and thus must be observed carefully. Lower temperatures might at least partly be compensated by the prudent use of AOB or other antimicrobial compounds. Still, if a particularly high antimicrobial efficacy is required, especially concerning immunocompromised persons, low temperatures and short durations of the washing cycle might not deliver a sufficient antimicrobial effect, even when an AOB-containing detergent is used.

## SUPPLEMENTAL MATERIAL

Click here for supplemental data file.

All supplemental data for this article are available online at http://www.microbialcell.com/researcharticles/2019a-bockmuehl-microbial-cell/.

## Abbreviations:

AOB: – activated oxygen bleach,
CDC: – Center for Disease Control and Prevention
MRSA: – multi-resistant Staphylococcus aureus,
QAC: – quaternary ammonium compounds,
RKI: – Robert-Koch-Institute,
TVC: – total viable counts.
