# Supplementary material for: Laundry and textile hygiene in healthcare and beyond
Source: Microb Cell. 2019 Jul 1;6(7):299–306. doi: 10.15698/mic2019.07.682 (PMC6600116; doi:10.15698/mic2019.07.682)
Supplement: Supplementary file 1 [file mic-06-299-s01.pdf]

microbial cell

CHECKLIST

www.microbialcell.com

HYGIENE IN HEALTHCARE
SPECIAL ISSUE

Checklist: Laundry and textile hygiene in healthcare

- ☐ Make sure that the factors combined in the laundry process are providing the suitable hygiene efficacy. Temperatures above 60°C ensure high antimicrobial effects even without chemical disinfectants. When lower temperatures are used, detergents with activated oxygen bleach or additives can be used.

- ☐ Consider the health status of the person's laundry is prepared for. In case of higher-risk-groups, such as babies, elderly persons, pregnant women or immunocompromised persons, a higher level of hygiene is required.

- ☐ The washing machine itself provides a considerable source of microorganisms and thus should be cleaned on a regular basis.

- ☐ Appliances for domestic do not always reach the chosen temperature. Therefore, professional washing machines should be considered for industrial or institutional purposes or whenever a certain hygiene efficacy is required.

This checklist represents the Supplemental Material of the publication „Laundry and textile hygiene in healthcare and beyond“ by Bockmühl *et al*. (2019)

Please cite as: Dirk P. Bockmühl, Jan Schages and Laura Rehberg (**2019**). Laundry and textile hygiene in healthcare and beyond. **Microbial Cell** 6(7): 299-306. doi: 10.15698/mic2019.07.682
